# Dual-energy CT for the detection of skull base invasion in nasopharyngeal carcinoma: comparison of simulated single-energy CT and MRI

**DOI:** 10.1186/s13244-023-01444-3

**Published:** 2023-05-24

**Authors:** Yang Zhan, Peng Wang, Yuzhe Wang, Yin Wang, Zuohua Tang

**Affiliations:** 1 Shanghai Institute of Medical Imaging, Fudan University, Shanghai, China; 2 Department of Radiology, Eye & ENT Hospital, Fudan University, 83 Fenyang Road, Shanghai, 200031 China; 3 Department of Radiology, Affiliated Hospital of Jiangnan University, Wuxi, China

**Keywords:** Dual-energy CT, Single-energy CT, Magnetic resonance imaging, Nasopharyngeal carcinoma, Skull base invasion

## Abstract

**Background:**

Skull base invasion in nasopharyngeal carcinoma (NPC) was shown to be a poor negative prognostic factor, and dual-energy CT (DECT) has heralded a new approach to detect this condition. The study aims to evaluate the value of DECT for detection of skull base invasion in NPC and compare the diagnostic performance of DECT with those of simulated single-energy CT (SECT) and MRI.

**Methods:**

The imaging findings of 50 NPC patients and 31 participants in control group which underwent DECT examinations were assessed in this retrospective study. The skull base invasions were evaluated using 5-point scale by two blind observers. ROC analysis, Mcnemar test, paired t test, weighted *K* statistics and intraclass correlation coefficient were performed to evaluate the diagnostic performance of simulated SECT, MRI and DECT.

**Results:**

Quantitative analysis of DECT parameters showed higher normalized iodine concentration and effective atomic number values in sclerosis and lower values in erosion than those in normal bones (both *p* < 0.05). Compared with simulated SECT and MRI, the diagnostic sensitivity for DECT was significantly improved from 75% (simulated SECT) and 84.26% (MRI) to 90.74% (DECT) (both *p* < 0.001), specificity from 93.23% and 93.75% to 95.31 (both *p* < 0.001), accuracy from 86.67% and 90.33% to 93.67%, and AUC from 0.927 and 0.955 to 0.972 (both *p* < 0.05), respectively.

**Conclusions:**

DECT demonstrates better diagnostic performance than simulated SECT and MRI for detecting skull base invasions in NPC, even those slight bone invasions in early stage, with higher sensitivity, specificity and accuracy.

**Graphical Abstract:**

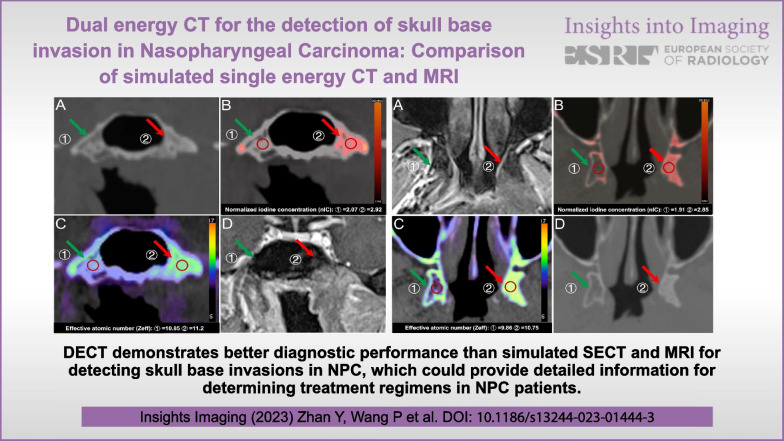

## Clinical relevance statement

DECT demonstrates better diagnostic performance than simulated SECT and MRI for detecting skull base invasions in NPC, which could provide detailed information for determining treatment regimens in NPC patients.

## Key points

- DECT appears to be more accurate in the diagnosis of skull base invasion when compared with SECT and MRI.
- DECT derived normalized iodine concentration (nIC) and effective atomic number (Zeff) values provide detailed quantitative parameters for diagnosing bone invasions.
- Different energy-dependent absorption behaviors of DECT helps detecting slight bone invasions with high sensitivity.

## Background

Nasopharyngeal carcinoma (NPC) is the most common type of head and neck squamous cell carcinoma; approximately, 129,100 new cases and 73,000 NPC deaths were estimated to occur annually worldwide [1]. NPC has a tendency to invade skull base above the nasopharynx [2] due to posterosuperior tumor extension [3]. Once skull base invasion is present, the tumor is divided into T3 [4], which means it is at an advanced stage, and could more easily lead to local recurrence and poor prognosis [5]. However, due to a wide range and complex anatomic structure of the skull base [6], the diagnostic sensitivity, specificity and accuracy of conventional imaging for detecting slight skull base invasion and extent in NPC are still not very high [7]. Therefore, searching for a more precise method to detect detailed skull base invasion in NPC has become increasingly urgent.

Conventional single-energy CT (SECT) and magnetic resonance imaging (MRI) are predominant diagnostic tools for evaluating tumor extension and related skull base invasion in NPC [8, 9]. SECT can provide intricate anatomical details of sclerotic and lytic lesions, but it cannot detect some of the initial invasions of bone marrow, especially those without cortex bone destroying [10]. Instead, MRI has been widely acknowledged to be sensitive when detecting early infiltration of tumors into the bone marrow [11]. However, peritumoral inflammation can mimic bone invasion even on MR images, leading to a number of false-positive findings [12, 13].

Dual-energy CT (DECT) has become a practical clinical imaging tool since 2006 [14] due to its advantage of distinguishing different materials by different energy-dependent absorption behaviors [15]. Several studies reported that DECT can be useful in differential diagnosis of various carcinomas [16, 17] and in predicting lymph node metastases [18] as well as proliferation levels [19] in cancers. Moreover, DECT can be used to characterize some specific substances, such as calcium, in human tissues [4, 20, 21]. Most importantly, different types of post-processing images derived from DECT, such as color-coded maps, can be exploited for better visual detectability of attenuation changes in the bone marrow [22]. The above advantages make it conceivable for DECT to detect altered marrow composition with higher accuracy than SECT [4, 20–22]. Therefore, the aim of this study was to evaluate the value of DECT for the detection of skull base invasions in NPC, especially for the slight bone invasions and precise extent of invasion, and compare the diagnostic performance of DECT with those of simulated SECT and MRI.

## Methods

### Study population

**Fig. 1:**
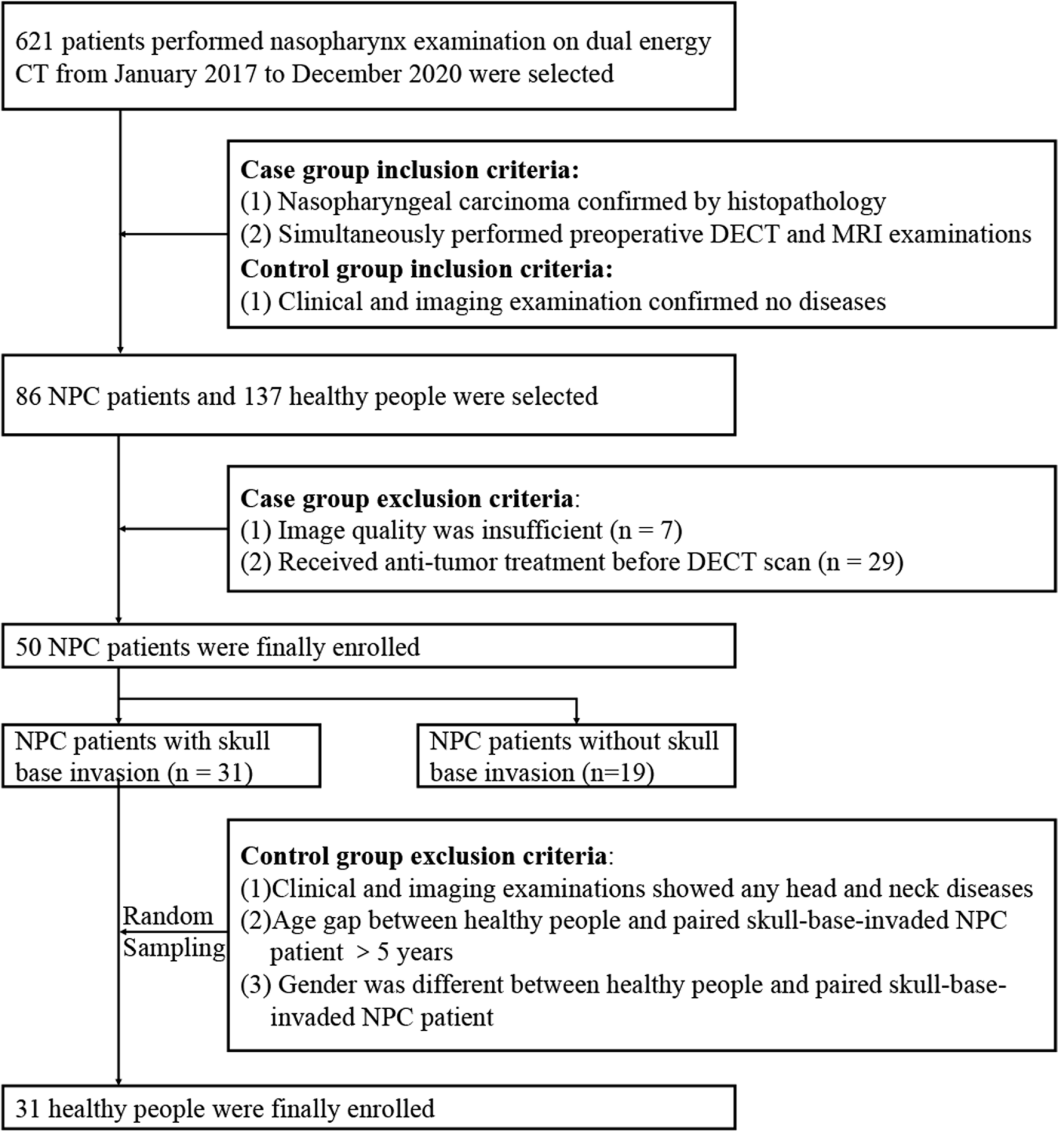
Flowchart of study design

The study was performed and reported according to the STARD reporting guidelines [23]. A total of 50 NPC patients from January 2017 to December 2020 were consecutively enrolled in this study (shown in Fig. 1). Recruitment was based on the following inclusion criteria: (1) all masses were confirmed by histopathology and (2) DECT and MRI were simultaneously performed before treatment (within 7 days). The exclusion criteria were as follows: (1) image quality and data were insufficient for evaluation and reconstruction and (2) patients had received any treatment before imaging examinations. To determine the differences of DECT parameters normalized iodine concentration (nIC) and effective atomic number (Zeff) values between skull base invasion and normal bones, thirty-one participants in control groups were also included in our study, and the inclusion criteria were as follows: (1) participants performed DECT examinations for suspected nasopharyngeal carcinoma due to persistently elevated Epstein-Barr virus DNA; (2) clinical and imaging examinations confirmed no head and neck diseases; (3) the age gap between subject and paired skull-base-invaded NPC patient was fewer than 5 years; (4) subject’s gender were the same as paired skull-base-invaded NPC patient; (5) subject was decided by random sampling.

### Imaging techniques

All DECT scans were performed using a 128-section CT scanner (Somatom Definition; Siemens Healthcare), based on peak kilovoltages of 80 kVp and 140 kVp. The DECT imaging parameters included a section collimation of 0.6 mm, gantry rotation time of 0.5 s, pitch of 0.6, matrix of 512 × 512, and field of view of 33 cm. Iodine concentration (IO) and Zeff images were obtained after administration of iopamidol 320 mg I/ml (Isovue; Bracco Diagnostics, Inc, Singen, Germany) using a split-bolus technique with administration of 45 ml iodized contrast at 4 ml/s for 11 s followed by a second bolus administration of 25 ml saline flushing at 2 ml/s for 13 s without delay. Commercial DE-equipped Workstation (Syngo; Siemens Healthcare) was utilized to acquire IO and Zeff images. The linear mixed image (Mix-0.3) can be reconstructed by using 30% from the 80 kVp and 140 kVp to simulate the conventional SECT. Tube current modulation was used for individual radiation dose optimization. Sinogram affirmed iterative reconstruction (SAFIRE) algorithm was used for data reconstruction with Kernel J30f medium smooth, and reconstruction plane was based on the sagittal plane. The mean volume CT dose index (CTDI_vol_) and dose length product (DLP) values were 7.8 ± 2.2 mGy and 266 ± 255 mGy.cm, respectively.

MRI examinations were performed on a 3T MR imaging scanner (Magnetom Verio; Siemens) using a 12-channel head coil. The MRI protocol consisted of axial fast spin-echo T1-weighted imaging (T1WI) (repetition time/echo time = 3840/91 ms) and axial fast spin-echo T2-weighted imaging (T2WI) (repetition time/echo time = 4000/99 ms). MR imaging parameters included an acquisition matrix of 640 × 592, FOV of 220 × 220 mm, number of averages of 2, thickness/gap of 6/0.6 mm, and parallel imaging acceleration factor of 2. Contrast-enhanced fs T1WI was performed after intravenous injection of 0.1 mmol/kg Gd-DTPA (Magnevist, Bayer Schering, Berlin, Germany) at a rate of 2 ml/s (TR/TE = 3840/91 ms), followed by a 20 ml 0.9% saline flush using a power injector.

### Imaging evaluation

All images were presented in random order, and simulated SECT, DECT and MRI images were independently reviewed by two experienced neuroradiologists (A with 10 years and B with 12 years of experience in head and neck radiology) who were unaware of clinical findings and images from other techniques. The final diagnosis was determined by consensus, and inconsistencies between readers A and B were resolved through the decision of another experienced radiologist (C with 21 years of experience in head and neck radiology). Firstly, radiologists were asked to observe the skull base invasions at six sites of each patient due to the differences of the amount of bone marrow between six sites, including the sphenoid body, clivus, bilateral pterygoid process and bilateral petrous apex. Secondly, regions of interest (ROI) were placed at the invaded bones on IO and Zeff images derived from DECT, and the iodine concentration and effective atomic number were calculated. Simultaneously, the same ROIs as the above invasions were also selected at normal bones in the control group. Simulated SECT images were evaluated first, followed 4 weeks and 8 weeks later by evaluation of DECT and MRI images, respectively. All images were presented in random order. The iodine concentration values were divided by the iodine concentration of the aorta to obtain the nIC. The values were averaged for statistical analysis. All invasions could be divided into lytic lesions and sclerotic lesions. According to the 8th AJCC staging system [4], TNM staging of nasopharyngeal carcinoma was performed based on clinical and imaging findings.

According to previous bone invasion studies [2, 24], a 5-point scale was used to evaluate skull base invasions by simulated SECT and DECT, and scores of > 3 were considered a positive diagnosis. To be more specific, lesions or iodine concentration separated from the bone at the base of skull were considered a score of 1 (definitely negative); lesions or iodine concentration just in contact with the skull base were considered a score of 2 (probably negative); erosion and sclerosis or iodine distribution of the skull base were considered a score of 3 (possibly positive); a score of 4 (probably positive); lesions that invaded bone marrow were considered a score of 5 (definitely positive).

For MRI images, the diagnosis of skull base invasion was mainly based on T1WI, contrast fs T1WI, and T2WI images. If the skull base adjacent to the tumor manifested as hypointense on T1WI and was enhanced on post-contrast T1WI, then the skull base invasion is considered positive. The extent of skull base invasion was evaluated using a 5-point scale scoring system [2, 24]: 1, definitely negative; 2, probably negative; 3, possibly positive; 4, probably positive; and 5, definitely positive.

Due to the difficulty in verifying skull base invasion pathologically, the reference standard of skull base invasion [14] was based on the combination of imaging features of all imaging techniques (simulated SECT, DECT and MRI). Firstly, all images were presented in random order, and two experienced neuroradiologists (D and E with 20 and 22 years of experience in head and neck radiology) comprehensively evaluated all images (simulated SECT, DECT and MRI) of each patient independently. Then, neuroradiologists D and E diagnosed the skull base invasions based on combination of all images in patients according to the following criteria: (1) bone destruction with decreased density/iodine concentration; (2) osteosclerosis with increased density/iodine concentration on simulated SECT/DECT images; (3) absence of fat signal in skull base on all pre-contrast T1WI; (4) contrast enhancement on the post-contrast T1WI with fat suppression. Moreover, the 6-month follow-up imaging was used to confirm the skull base invasion in NPC, and skull base invasions that persisted or became larger in images after 6 months confirmed the existence of bone invasion. Lastly, the final diagnosis was determined by consensus.

### Statistical analysis

The average age of patients, nIC and Zeff values of skull base invasions are presented as the mean ± SD. The comparison of sensitivity and specificity among simulated SECT, MRI and DECT was performed using the Mcnemar test. The comparison of nIC and Zeff values between the NPC and control groups was performed using paired t tests.

ROC curves and the area under the curve (AUC) for simulated SECT, MRI and DECT were compared using Delong’s test. A 95% confidence interval was calculated using ROC analysis.

The inter-reader reproducibility for scores in simulated SECT, MRI and DECT images was evaluated using weighted *K* statistics [25]. A 95% confidence interval was calculated for each *K*. The agreement of nIC and Zeff value measurements between two readers was calculated using the intraclass correlation coefficient (ICC). ICC values of > 0.75 were considered indicative of good agreement.

Statistical analyses were performed in Excel 2016 (Microsoft), MedCalc statistical software (Version 15.2.2; MedCalc Software), and SAS (Version 9.2; SAS Institute Inc.). *p* values < 0.05 were considered statistically significant.

## Results

### Demographic characteristics and skull base involvement in NPC patients

The clinical characteristics and imaging findings of all 50 NPC patients (male: 37; female: 13; average age: 52.52 ± 12.02) and 31 subjects in the control group (male: 19; female: 12; average age: 50.55 ± 15.93) were assessed. Of all 50 NPC patients, TNM staging based on the 8th edition of the AJCC staging system included T1 (9/50, 18%), T2 (7/50, 14%), T3 (19/50, 38%), T4 (15/50, 30%), N0 (16/50, 32%), N1 (15/50, 30%), N2 (12/50, 24%), N3 (7/50, 14%), M0 (47/50, 94%), and M1 (3/50, 6%).

### Comparison of DECT parameters between invaded skull base in the NPC group and normal skull base in the control group

**Fig. 2:**
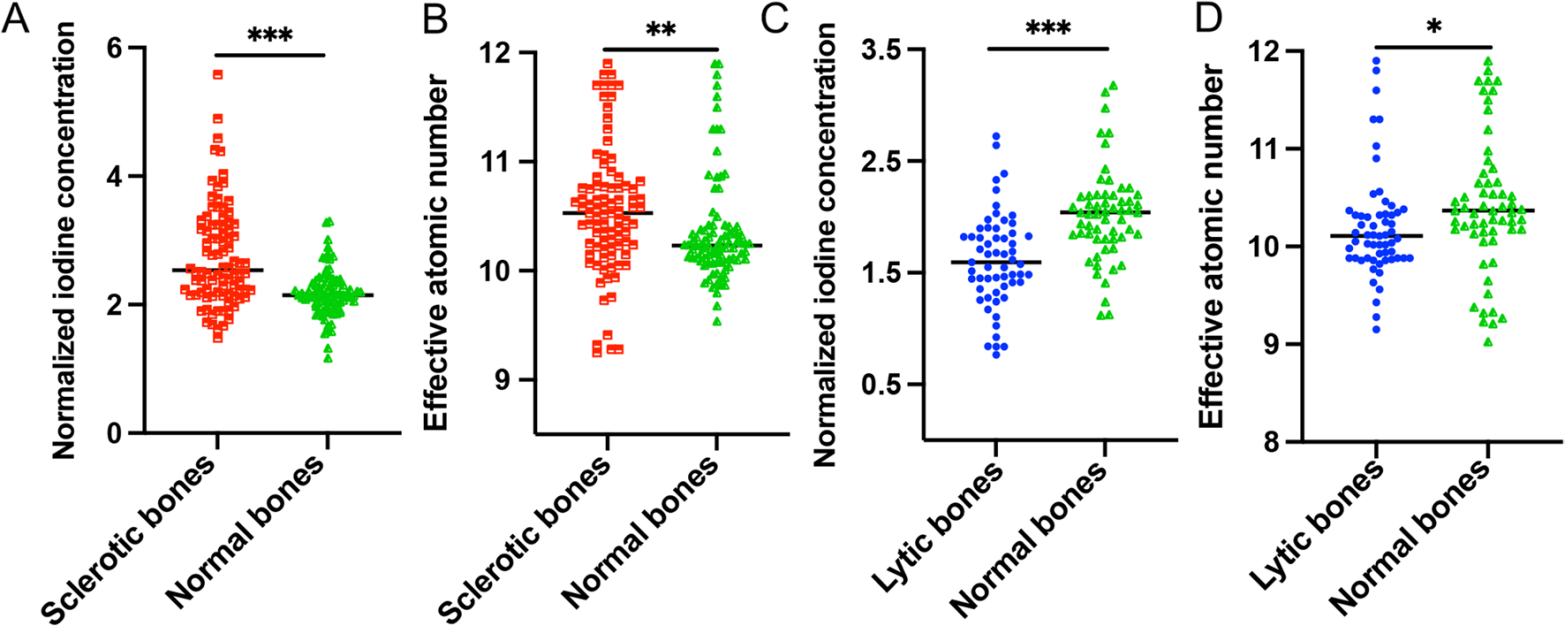
Comparison of quantitative parameters on DECT between invaded bones and normal bones in NPC patients. The normalized iodine concentration (**A**, nIC) and effective atomic number (**B**, Zeff) values of sclerotic bones are significantly higher than those of normal bones, respectively (both *p* < 0.01). The nIC (**C**) and Zeff (**D**) values of lytic bones are significantly lower than those of normal bones, respectively (both *p* < 0.05)

Table 1 shows 149 lesions at 108 skull base invasion sites (49 sites with sclerotic lesions, 18 sites with lytic lesions, and 41 sites with both sclerosis and lytic lesions) in 31 NPC patients. As shown in Fig. 2, The quantitative analysis of DECT parameters showed that nIC and Zeff values at sclerotic bones in the NPC group were significantly higher than those at normal bones in the control group (both *p* < 0.01), and nIC and Zeff values at the lytic bones were significantly lower than those at normal bones (both *p* < 0.05).

**Table 1:** Quantitative dual-energy parameters between invaded bones in NPC patients and normal bones in control group

Parameter	Normalized iodine concentration				Effective atomic number			
	Invaded bones^a^	Normal bones^a^	*t* value^c^	*p* value^c^	Invaded bones^b^	Normal bones^b^	*t* value^c^	*p* value^c^
All lesions (*n* = 149)								
Sclerotic lesions (*n* = 90)	2.8 ± 0.8	2.2 ± 0.4	8.0	< 0.0001*	10.6 ± 0.6	10.4 ± 0.5	2.8	0.007*
Lytic lesions (*n* = 59)	1.6 ± 0.4	2.0 ± 0.4	8.1	< 0.0001*	10.2 ± 0.5	10.4 ± 0.7	2.2	0.031*
Sphenoid body (*n* = 39)								
Sclerotic lesions (*n* = 26)	2.8 ± 1.0	2.2 ± 0.4	3.3	0.003*	10.8 ± 0.6	10.4 ± 0.5	2.8	0.011*
Lytic lesions (*n* = 13)	1.6 ± 0.5	2.4 ± 0.5	4.8	< 0.0001*	10.3 ± 0.5	10.5 ± 0.5	0.9	0.375
Clivus (*n* = 28)								
Sclerotic lesions (*n* = 13)	2.2 ± 0.6	1.8 ± 0.3	2.9	0.013*	10.4 ± 0.6	10.3 ± 0.6	0.4	0.706
Lytic lesions (*n* = 15)	1.2 ± 0.3	1.6 ± 0.3	6.3	< 0.0001*	10.0 ± 0.6	10.4 ± 0.9	2.1	0.058
Left pterygoid process (*n* = 28)								
Sclerotic lesions (*n* = 17)	3.2 ± 0.6	2.3 ± 0.4	7.2	< 0.0001*	10.5 ± 0.6	10.4 ± 0.5	1.1	0.295
Lytic lesions ( = 11)	1.9 ± 0.4	2.2 ± 0.3	3.4	0.007*	10.4 ± 0.6	10.5 ± 0.7	0.4	0.724
Right pterygoid process (*n* = 19)								
Sclerotic lesions (*n* = 12)	3.1 ± 0.8	2.4 ± 0.3	4.1	0.002*	10.4 ± 0.7	10.1 ± 0.2	1.4	0.198
Lytic lesions (*n* = 7)	1.7 ± 0.2	2.1 ± 0.1	8.3	0.0002*	10.1 ± 0.2	10.1 ± 0.5	0.1	0.946
Left Petrous apex (*n* = 22)								
Sclerotic lesions (*n* = 13)	2.5 ± 0.6	2.1 ± 0.3	3.4	0.005*	10.3 ± 0.4	10.4 ± 0.5	0.4	0.701
Lytic lesions (*n* = 9)	1.6 ± 0.2	1.9 ± 0.2	3.6	0.007*	10.4 ± 0.6	10.6 ± 0.7	0.8	0.469
Right Petrous apex (*n* = 13)								
Sclerotic lesions (*n* = 9)	2.4 ± 0.4	2.3 ± 0.1	1.3	0.238	10.7 ± 0.5	10.4 ± 0.5	2.8	0.023*
Lytic lesions (*n* = 4)	1.9 ± 0.3	2.0 ± 0.2	5.7	0.608	10.2 ± 0.2	10.6 ± 0.4	1.8	0.180

*Significant difference compared with nIC of normal bones (*p* < 0.05)

^a^Value of normalized iodine concentration (nIC)

^b^Value of effective atomic number (Zeff)

^c^As determined with paired *t* test

### Diagnostic performance of simulated SECT, MRI, and DECT for detection of skull base invasion in NPC

**Fig. 3:**
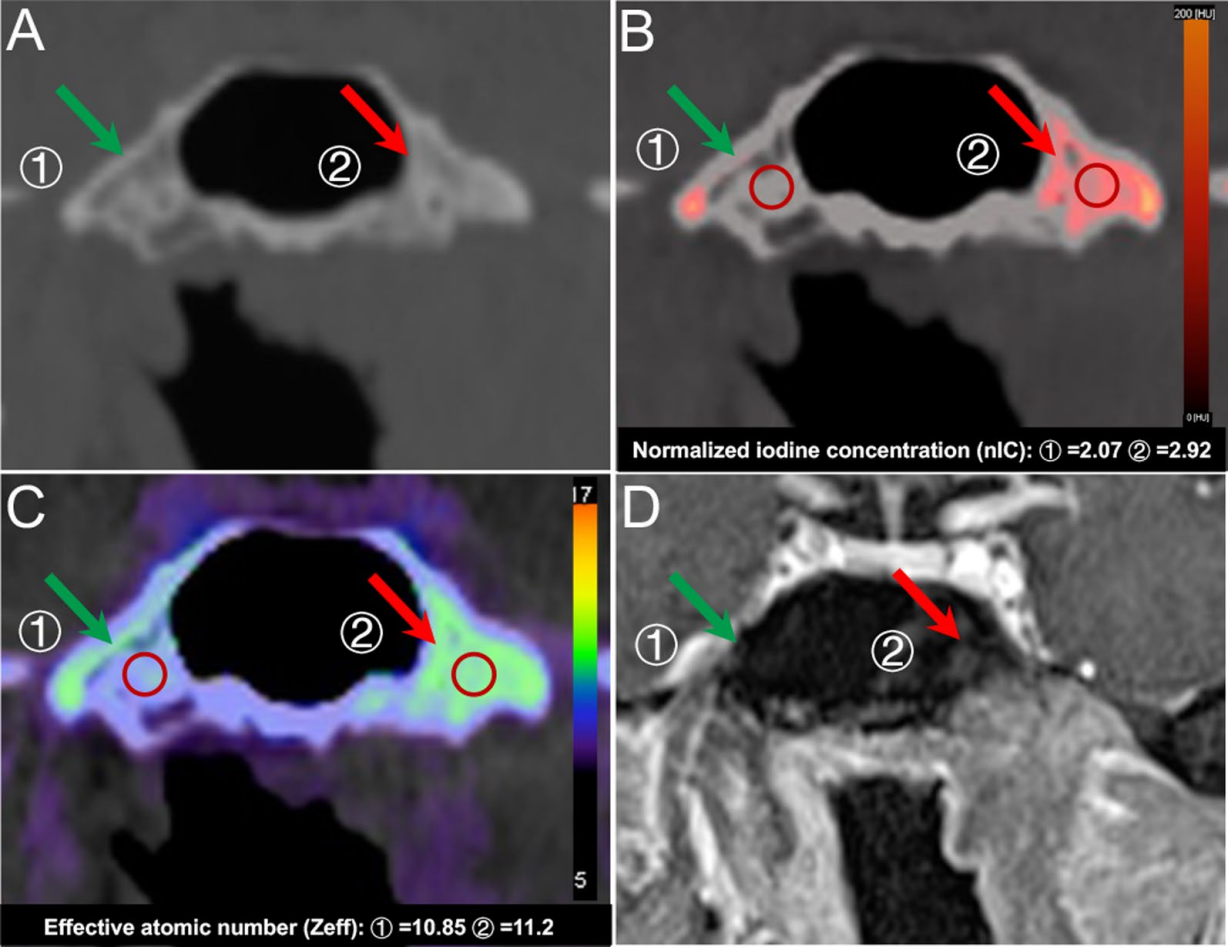
Suspicious false-negative finding for skull base invasions by SECT images in a 54-year-old man with NPC. **A** The axial SECT images (bone window) show suspicious sclerosis at the left sphenoid body (red arrow). The DECT images (**B**, IO image; **C**, Zeff image) directly show marked skull base invasions with higher iodine concentrations than right sphenoid body (green arrow). **D** The MRI images (fat-suppressed T1-weighted image after gadolinium administration) confirm the same extent of bone invasions as the DECT images with contrast enhancement

**Fig. 4:**
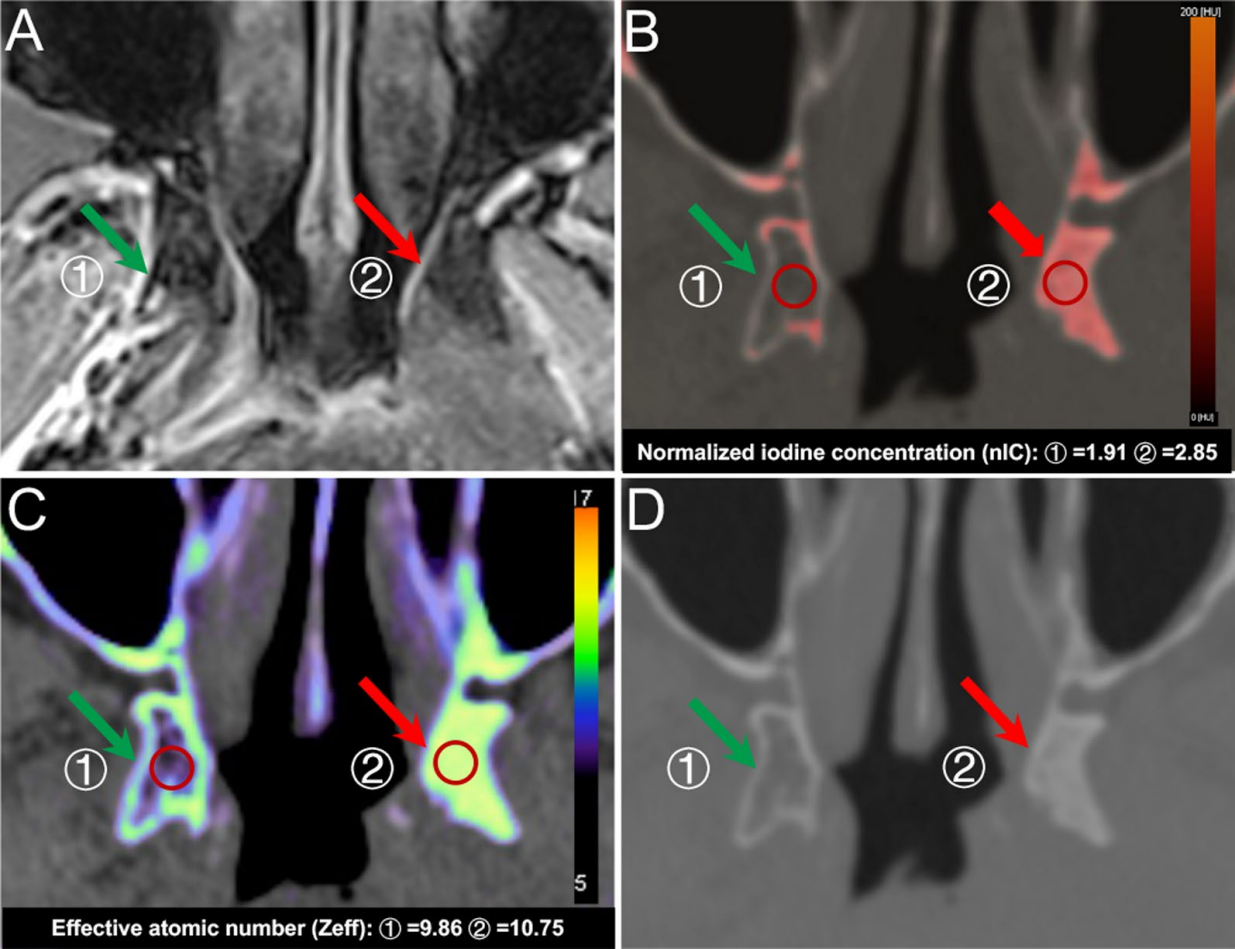
False-negative findings for skull base invasions by MRI in a 43-year-old man with NPC. **A** The axial MRI images (fat-suppressed T1-weighted image after gadolinium administration) do not show obvious abnormal contrast enhancement at the left pterygoid process (red arrow). The DECT images (**B**, IO image; **C**, Zeff image) directly show marked bone invasions with higher iodine concentrations than right pterygoid process (green arrow). **D** The SECT images (bone window) confirm the invasions with sclerosis at the left pterygoid process like the DECT images

Of all 108 skull base invasion sites, 81 (75.0%) were correctly shown on SECT, 91 (84.3%) on MRI, and 98 (90.7%) on DECT according to 5-point scale scoring system [2, 24] (shown in Table 2). In terms of the other 192 sites without skull base invasion, 179 sites (93.2%) were correctly diagnosed by SECT, 180 sites (93.8%) by MRI, and 183 sites (95.3%) by DECT. In particular, for slight bone invasions (score 3), five false-negative findings by simulated SECT and ten false-negative findings by MRI were recognized by DECT. Additionally, Figs. 3 and 4 show representative suspicious false-negative findings by SECT and representative false-negative findings by MRI, respectively.

**Table 2:** Comparison of imaging findings of skull base invasions between SECT, MRI, and DECT in NPC patients

Parameter	TP^a^	TN^a^	FN^a^	FP^a^	Sensitivity (%)	Specificity (%)	PPV (%)	NPV (%)
All sites (*n* = 300)								
SECT	81	179	27	13	75.00 (65.7–82.8)	93.23 (88.7–96.3)	86.2	86.9
MRI	91	180	17	12	84.26 (76.0–90.6)	93.75 (89.3–96.7)	88.3	91.4
DECT	98	183	10	9	90.74 (83.6–95.5)	95.31 (91.3–97.8)	96.3	91.3
*p* value^b1^					< 0.001*	< 0.001*		
*p* value^b2^					< 0.001*	< 0.001*		
Sphenoid body (*n* = 50)								
SECT	22	20	6	2	78.57 (59.0–91.7)	90.91 (70.8–98.9)	85.7	88.1
MRI	25	21	3	1	89.29 (71.8–97.7)	95.45 (77.2–99.9)	96.2	87.5
DECT	26	21	2	1	92.86 (76.5–99.1)	95.45 (77.2–99.9)	96.3	91.3
*p* value^c1^					0.04*	0.09		
*p* value^c2^					0.21	< 0.05*		
Clivus (*n* = 50)								
SECT	14	26	7	3	66.67 (43.0–85.4)	89.66 (72.6–97.8)	82.4	78.8
MRI	17	27	4	2	80.95 (58.1–94.6)	93.10 (77.2–99.2)	89.5	87.1
DECT	18	27	3	2	85.71 (63.7–97.0)	93.10 (77.2–99.2)	90.0	90.0
*p* value^c1^					0.03*	0.20		
*p* value^c2^					0.08	0.14		
Left pterygoid process (*n* = 50)								
SECT	15	30	4	1	78.95 (54.4–93.9)	96.77 (88.3–99.9)	93.8	88.2
MRI	17	29	2	2	89.47 (66.9–98.7)	93.55 (78.6–99.2)	89.5	93.5
DECT	17	30	2	1	89.47 (66.9–98.7)	96.77 (83.3–99.9)	94.4	93.7
*p* value^c1^					0.39	.07		
*p* value^c2^					< 0.01*	.03*		
Right pterygoid process (*n* = 50)								
SECT	10	35	3	2	76.92 (46.2–95.0)	94.59 (81.8–99.3)	83.3	92.1
MRI	11	36	2	1	84.62 (54.6–98.1)	97.30 (85.8–99.9)	91.7	94.7
DECT	12	36	1	1	92.31 (64.0–99.8)	97.30 (85.8–99.9)	92.3	97.3
*p* value^c1^					0.23	.05		
*p* value^c2^					0.15	.03*		
Left Petrous apex (*n* = 50)								
SECT	12	30	4	4	75.00 (47.6–92.7)	88.24 (72.5–96.7)	75.0	88.2
MRI	14	31	2	3	87.50 (61.7–98.4)	91.18 (76.3–98.1)	82.4	93.9
DECT	15	31	1	3	93.75 (69.8–99.8)	91.18 (76.3–98.1)	83.3	96.9
*p* value^c1^					0.25	< 0.01*		
*p* value^c2^					0.13	0.02*		
Right Petrous apex (*n* = 50)								
SECT	8	38	3	1	72.73 (39.0–94.0)	97.44 (86.5–99.9)	88.9	92.7
MRI	7	36	4	3	63.64 (30.8–89.1)	94.87 (82.7–99.4)	77.8	90.2
DECT	10	38	1	1	90.91 (58.7–99.8)	97.44 (86.5–99.9)	90.9	97.4
*p* value^c1^					0.27	0.03*		
*p* value^c2^					0.36	0.08		

*TP* true positive findings, *TN* true-negative findings, *FN* false-negative findings, *FP* false-positive findings, *PPV* positive predictive value, *NPV* negative predictive value

*Significant difference compared with DECT (*p* < 0.05)

^a^Numbers of findings

^b^As determined with the McNemar test

^c^According to the generalized linear mixed model that accounted for the multiple observations within patients

^1^SECT versus DECT

^2^MRI versus DECT

**Fig. 5:**
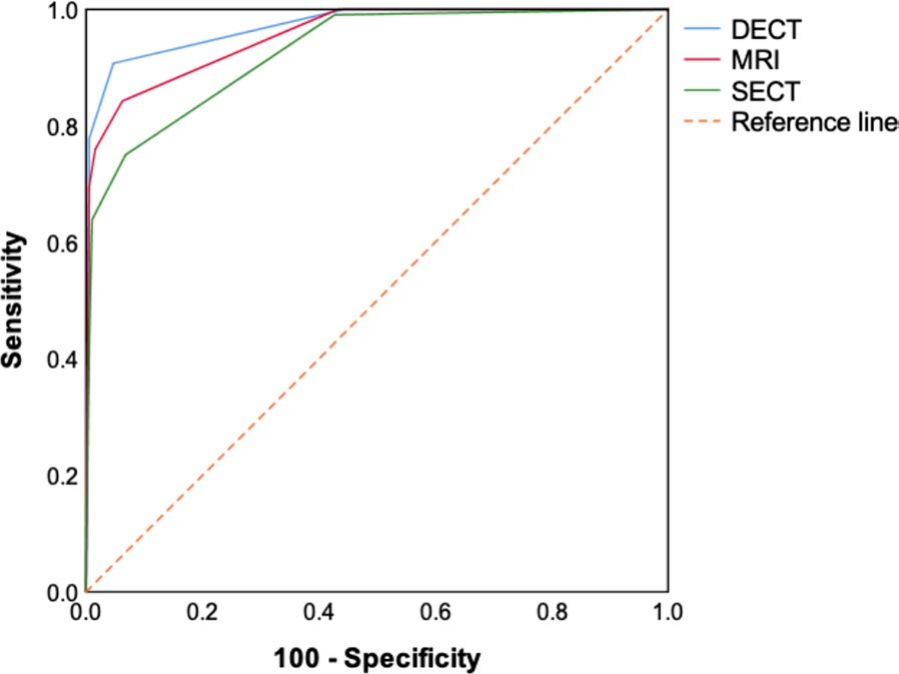
ROC curves with SECT, MRI, and DECT for the diagnosis of skull base invasions. The AUCs for SECT, MRI, and DECT were 0.927, 0.955, and 0.972, respectively. (0.927 vs. 0.972, *p* < 0.001; 0.955 vs. 0.972, *p* = 0.028)

As shown in Table 2, the diagnostic sensitivity and specificity at overall sites of DECT were significantly higher than that of simulated SECT and MRI (both *p* < 0.001) according to 5-point scale scoring system [2, 24]. Compared with SECT and MRI, the diagnostic accuracy of overall sites for DECT was improved from 86.67% (simulated SECT) and 90.33% (MRI) to 93.67% (DECT). The comparison of ROC curves by using Delong’s test (shown in Fig. 5) demonstrated that the AUC of DECT for detecting skull base invasion in NPC was (95% CI 0.95–0.99) significantly higher than MRI (95% CI 0.93–0.98; *p* < 0.001) and simulated SECT (95% CI 0.89–0.95; *p* < 0.05), respectively.

#### Interobserver reproducibility

As shown in Table 3, weighted kappa values were used to assess the inter-reader agreement of the five observers for simulated SECT, MRI, and DECT. The kappa value at all sites for DECT (*K* = 0.84–0.93) was higher than that for simulated SECT (*K* = 0.77–0.89) and MRI (*K* = 0.83–0.92). The intraclass correlation coefficients of nIC (ICC = 0.812; 95% CI, 0.770–0.847) and Zeff (ICC = 0.830; 95% CI 0.791–0.862) values derived from DECT between the two readers showed good interobserver agreement for the evaluation of the quantitative parameters.

**Table 3:** Interobserver agreement in the evaluation of skull base invasions for SE-CT, MRI, and DECT in NPC patients

Parameter and reader	SECT images	MR images	DECT images
All sites (*n* = 300)			
A versus B	0.77 (0.73–0.81)	0.83 (0.79–0.86)	0.84 (0.81–0.88)
A versus C	0.88 (0.85–0.91)	0.91 (0.89–0.94)	0.93 (0.91–0.95)
B versus C	0.89 (0.86–0.92)	0.92 (0.89–0.95)	0.91 (0.89–0.94)
D versus E	0.92 (0.89–0.94)		
Sphenoid body (*n* = 50)			
A versus B	0.80 (0.71–0.90)	0.83 (0.75–0.91)	0.88 (0.81–0.94)
A versus C	0.89 (0.82–0.96)	0.92 (0.87–0.97)	0.93 (0.88–0.98)
B versus C	0.92 (0.85–0.98)	0.91 (0.84–0.98)	0.94 (0.89–0.99)
D versus E	0.91 (0.85–0.97)		
Clivus (*n* = 50)			
A versus B	0.78 (0.69–0.87)	0.84 (0.75–0.92)	0.85 (0.78–0.92)
A versus C	0.89 (0.83–0.96)	0.92 (0.86–0.98)	0.93 (0.88–0.98)
B versus C	0.89 (0.82–0.96)	0.92 (0.85–0.98)	0.92 (0.88–0.96)
D versus E	0.94 (0.89–0.98)		
Left pterygoid process (*n* = 50)			
A versus B	0.76 (0.65–0.86)	0.80 (0.71–0.90)	0.83 (0.75–0.91)
A versus C	0.88 (0.80–0.96)	0.91 (0.84–0.97)	0.93 (0.87–0.98)
B versus C	0.86 (0.78–0.94)	0.90 (0.83–0.97)	0.90 (0.83–0.97)
D versus E	0.93 (0.87–0.99)		
Right pterygoid process (*n* = 50)			
A versus B	0.75 (0.62–0.88)	0.81 (0.72–0.91)	0.81 (0.72–0.91)
A versus C	0.89 (0.80–0.97)	0.88 (0.80–0.96)	0.91 (0.85–0.98)
B versus C	0.83 (0.72–0.94)	0.93 (0.87–0.99)	0.90 (0.82–0.98)
D versus E	0.89 (0.83–0.96)		
Left Petrous apex (*n* = 50)			
A versus B	0.76 (0.68–0.85)	0.83 (0.74–0.92)	0.80 (0.71–0.89)
A versus C	0.88 (0.81–0.96)	0.90 (0.84–0.97)	0.94 (0.89–0.99)
B versus C	0.88 (0.82–0.95)	0.93 (0.86–0.99)	0.86 (0.78–0.95)
D versus E	0.90 (0.83–0.97)		
Right Petrous apex (*n* = 50)			
A versus B	0.74 (0.63–0.84)	0.82 (0.73–0.92)	0.84 (0.76–0.93)
A versus C	0.85 (0.76–0.93)	0.91 (0.84–0.98)	0.93 (0.87–0.99)
B versus C	0.88 (0.81–0.97)	0.91 (0.84–0.98)	0.91 (0.85–0.98)
D versus E	0.92 (0.83–0.99)		

Data are weighted *K* values with 95% confidence intervals. Weighted *K* values of less than 0 indicate poor agreement; 0–0.2, slight agreement; 0.21–0.4, fair agreement; 0.41–0.6, moderate agreement; 0.61–0.8, substantial agreement; and 0.81–1, almost perfect agreement

## Discussion

This is the first study to evaluate whether the DECT could more accurately distinguish skull base invasion in NPC than simulated SECT and MRI. Our findings demonstrated that DECT showed better diagnostic performance for skull base invasions in NPC than simulated SECT and MRI, even those slight bone invasions in early stage and precise extent, characterized by higher sensitivity, specificity and accuracy than simulated SECT and MRI.

Compared with the sensitivity of simulated SECT for detecting skull base invasion in NPC, our study demonstrated that of DECT was significantly higher according to 5-point scale scoring system [2, 24] (*p* < 0.001). Additionally, lower false-negative rate of DECT than SECT was also observed in our study, consistent with previous SECT studies [10, 11]. Most importantly, for slight bone invasions (score 3), five false-negative findings by simulated SECT and ten false-negative findings by MRI were recognized by DECT. Hence, DECT was more sensitive than SECT for detecting skull base invasions, even these slight invasions in early stage. In addition, two characteristics of DECT (quantitative Zeff and nIC value) could account for its excellent diagnostic sensitivity for detecting skull base invasion. First, in our study, quantitative analysis of DECT parameters demonstrated that sclerosis showed higher Zeff values, and erosion bones showed lower Zeff values than normal bones. As denoted by Goo et al. [26], Zeff value is a quantitative parameter for different tissues via the differences in atomic numbers. In histopathology, when malignant tumor invades bone, the normal bone is substituted by tumor tissues at erosion bones [27], and tumor induces bony proliferative reaction at sclerosis [28]. As a result, the bone content deviation could be apparent in skull base invasion presented as abnormal Zeff value, consistent with the findings of Zeff in previous study [27]. Therefore, Zeff value may be a sensitive indicator of bone invasions in NPC. Moreover, our study showed compared with normal bones, DECT derived nIC values were significantly higher at sclerosis. In a previous study by Pang et al. [29], IC value was considered to be another quantitative parameter to indicate microvessel density in tissues. In histopathology, tumor cells invaded bone marrow at the early stage via circulation [27], then growth of tumor cells induced vascular network forming, leading to the markedly high expression of microvessel density in NPC [30]. As a result, high nIC value may be an indicator of invaded bone marrow in the early stage, consistent with a previous DECT study [31]. Consequently, nIC value may be beneficial for distinguishing slight bone invasions in the early stage. Thus, DECT and quantitative parameters, especially Zeff and nIC value, may facilitate higher diagnostic sensitivity of slight skull base invasion in the early stage and development of more precise treatment plans than SECT in NPC.

In comparison with the specificity of simulated SECT for detecting skull base invasion in NPC, our study demonstrated that of DECT was significantly higher on the basis of 5-point scale scoring system [2, 24] (*p* < 0.001), respectively. Moreover, our study also showed the overdiagnosis rate of DECT was lower than SECT. The overdiagnosis findings on SECT may be due to the low contrast resolution and beam-hardening artifacts produced by bone cortex [2]. In addition, in a previous study by Goo et al. [26], DECT could simultaneously increase iodine contrast-to-noise ratio and decrease metal or beam-hardening artifacts. As a result, DECT could reduce rate of overdiagnosis caused by artifacts on SECT, consistent with a previous study by B Li et al. [32]. Hence, the higher diagnostic specificity of DECT than SECT for detecting skull base invasion indicated that DECT could provide more detailed information for accurately determining chemotherapy plans in NPC.

In contrast to the AUC of SECT for detecting skull base invasion in NPC, that of DECT was significantly higher based on 5-point scale scoring system [2, 24] in our study (*p* < 0.001). Additionally, Fig. 3 shows that DECT precisely recognized the extent of invasion in NPC, whereas SECT showed a suspicious extent of invasion, consistent with several previous studies [22, 33–35]. Furthermore, according to clinical TNM staging criteria [4, 21], once skull base invasion is present, the tumor is divided into T3. Hence, the better diagnostic performance of DECT for detection of skull base invasion than SECT plays an important role in determining the extent of abnormal changes of bone and precise TNM staging in NPC. Therefore, compared with SECT, this comparative protocol confirmed that DECT has the ability to improve the diagnostic accuracy of skull base invasion and TNM staging in NPC, and that is essential to determine accurate planning target volume given in radiotherapy and reduce the risk of recurrence.

Compared with the false-positive rate of MRI for detecting skull base invasion in NPC, our study showed that of DECT was significantly lower in light of 5-point scale scoring system [2, 24]. In histopathology, the presence of fibrous stroma is a predominant feature of bone invasion, resulting in hypointense in T1 weighted image [36]; as a consequence, it may display similar diagnostic features as the reactive inflammation [13] and lead to false-positive findings on MRI, consistent with previous studies using MRI to detect bone invasions [37–40]. Additionally, Fig. 4 also showed one representative false-negative finding by MRI (one sclerotic lesion in the left pterygoid process was successfully recognized by SECT and DECT but undiagnosed by MRI), similar to a previous MRI study by Le et al. [41]. Therefore, these false-positive and false-negative findings on MRI with regard to bone invasions were still a serious challenge, and a new, better diagnostic protocol needs to be explored in our study. Furthermore, our study confirmed that compared with MRI, DECT demonstrated higher diagnostic sensitivity (*p* < 0.001), specificity (*p* < 0.001), and AUC (p = 0.028). Our results were in good agreement with several previous studies [41, 42], in which DECT uses the rapid switching of high-tube and low-tube voltages to provide precisely registered high-energy and low-energy datasets for material decomposition, which may make it easier to identify subtle bone involvement in NPC. Thus, DECT could help reducing false-positive and false-negative rates and improving diagnostic performances of MRI for detecting skull base invasion in NPC, consistent with previous studies [24, 41]. Hence, compared with MRI, DECT could be used as a more ancillary diagnostic tool to determine precise extent of tumor invasions, make individualized treatment plans, and further improve prognosis in NPC.

## Limitations

There are several limitations in the current study. First, there was a lack of surgical or pathological confirmation due to the difficulty in obtaining bone tissues; the reference standard of skull base invasion was based on combination of imaging features of all imaging techniques and 6-month follow-up. Second, this study is a retrospective analysis and, thus, is subject to a variety of biases such as potential selection bias inherent in convenience sampling [43]. Third, there was the potential for confounding bias of interobserver error, such as display settings. Last, unenhanced DECT scanning was not applied in this study due to a 30% radiation dose increase [44].

## Conclusions

This comparative analysis shows that DECT demonstrates better diagnostic performance than simulated SECT and MRI according to 5-point scale scoring system, with higher sensitivity, specificity, and accuracy, revealing that DECT is a potential imaging tool to accurately detect invaded bones in NPC, even those slight bone invasions in the early stage, which could provide detailed information for determining therapeutic strategies.

## Data Availability

The datasets used and/or analyzed during the current study are available from the corresponding author upon reasonable request.

## Abbreviations

AUC: Area under the curve
CTDI_vol_: Volume CT dose index
DECT: Dual-energy CT
DLP: Dose length product
fs T1WI: Fat suppression T1-weighted imaging
ICC: Intraclass correlation coefficient
IO: Iodine concentration
NIC: Normalized iodine concentration
NPC: Nasopharyngeal carcinoma
ROI: Regions of interest
SAFIRE: Sinogram affirmed iterative reconstruction
SECT: Single-energy CT
T2WI: T2-weighted imaging
Zeff: Effective atomic number
